# Locked and Unlocked Nucleosides in Functional Nucleic Acids

**DOI:** 10.3390/molecules16064511

**Published:** 2011-05-27

**Authors:** Holger Doessing, Birte Vester

**Affiliations:** Nucleic Acid Center, Department of Biochemistry and Molecular Biology, University of Southern Denmark, Campusvej 55, Odense M 5230, Denmark; E-Mail: holgerdoessing@gmail.com (H.D.)

**Keywords:** locked nucleic acid (LNA), unlocked nucleic acid (UNA), aptamer, ribozyme, deoxyribozymes (DNAzyme)

## Abstract

Nucleic acids are able to adopt a plethora of structures, many of which are of interest in therapeutics, bio- or nanotechnology. However, structural and biochemical stability is a major concern which has been addressed by incorporating a range of modifications and nucleoside derivatives. This review summarizes the use of locked nucleic acid (LNA) and un-locked nucleic acid (UNA) monomers in functional nucleic acids such as aptamers, ribozymes, and DNAzymes.

## 1. Introduction

Ribozymes, DNAzymes, and aptamers, collectively referred to as ‘functional nucleic acids’, are RNA or DNA structures with sequence-specific folds. These functional nucleic acids achieve their tertiary folds and activity through a combination of different molecular interactions and motifs: Hydrogen bonds, hydrophobic interactions, van der Waals forces, canonical and non-canonical base pairs, base stacking, coaxial stacking, tetraloops, G-quadruplexes, and metal ion coordination [1,2]. However, the use of nucleic acids in therapeutics and bio- and nanotechnologies is troubled by denaturation and/or biodegradation of the nucleic compounds. Non-natural nucleosides may offer improved half-life *in vivo*, better structural stability, or novel interacting groups. 2’-modified ribonucleoside analogues have attracted interest, as modification to this position on the ribose often confers improved nuclease resistance and allows fine-tuning of the helical structure. ‘Locked’ ribonucleoside analogues [3,4,5] in particular have gained interest over the last decade, and the recent advent of the conceptually opposite ‘unlocked’ nucleoside analogues [6,7] have expanded the biochemists’ tool set even further.

## 2. Locked Nucleic Acids

### Structure and Properties

**Figure 1:**
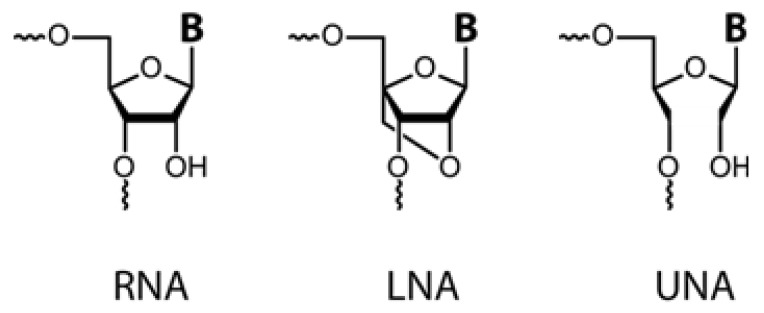
Structures of RNA, LNA, and UNA monomers. LNA is ‘locked’ in the RNA-like C3’-endo conformation due to the methylene linker. UNA lacks the C2’-C3’ bond and is therefore acyclic and ‘unlocked’.

A locked nucleic acid (LNA) is a ribonucleoside homologue that features a 2’-O,4’-C-methylene linker or bridge (Figure 1) [3,4,5,8]. This locks the ribose moiety in the C3’-endo conformation and makes LNA an RNA mimic. In duplexes, LNA causes a local re-organization of the phosphate backbone, including pucker steering of its neighbouring 3’ nucleotides towards the A-form, and replacing every third residue or more with its corresponding LNA moiety yields a near-canonical A-form heteroduplex [9]. By pre-arranging the nucleobases for better stacking, the enthalpy loss is increased upon duplex formation, while simultaneously reducing entropy loss [10]. This translates into very high thermal stability of LNA-modified duplexes. Introduction of LNA into the DNA strand of a DNA:RNA duplex has been shown to increase the T_m_ between 1–8 °C per each LNA moiety incorporated (compared to the unmodified duplex) [3,11,12,13]. Modification of the RNA strand in a similar heteroduplex has led to increases in T_m_ of no less than 2–10 °C per LNA moiety [3,11,12,13,14,15]. This property of LNA has been put to use in applications where high affinity is desirable, such as gene silencing [16], modulation of RNA splicing [17,18,19,20], RNA interference [21], molecular beacon probes [22,23,24,25], miRNA detection reviewed in [26] and DNAzymes [27,28,29,30,31,32,33,34,35,36,37].

The toxicity of LNA has been assessed in several studies: No changes were observed in body temperature and histological preparations of brains from rats injected with antisense LNA oligonucleotides [38], and mice repeatedly dosed with an LNA antisense oligomer at 1–2 mg/kg [39], 5 mg/kg [40], or 25 mg/kg [17,41] showed no serum liver cytotoxicity markers at this level. Stein *et al.* [42] maintained LNA antisense-mediated gene silencing in cell cultures for >240 days without observing any toxic side-effects. Swayze *et al.* [43] found, however, that mice treated repeatedly with LNA oligonucleotides experienced significant weight loss and were adversely affected, as judged from enzymatic assays and histopathological analyses of liver sections. Given the strong base-pairing potential of LNA-modified antisense oligonucleotides off-target effects might explain these results. Santaris Pharma A/S currently has LNA-based drugs against e.g., hepatitis C or tumor growth, in clinical trials [44,45].

One of the motivations for employing LNA is its stability towards nucleases. Short interfering RNAs (siRNA) are rapidly degraded by blood ribonucleases, but introduction of two LNA moieties at both 3’ termini has been shown to offer protection in human serum beyond 6 hours, a 4-fold improvement over the unmodified siRNA. Replacing one strand with a DNA/LNA mix-mer further improved stability beyond 48 h. Similar results were obtained in 10% foetal bovine serum and 100% mouse serum [46], as well as in live mouse blood [47] and with similar oligo designs [48].

Deoxyribonuclease activity in blood is generally believed to be shared among deoxyribonuclease I (DNase I), DNase II, and phosphodiesterase I with the former being the main component. Two or more LNA moieties towards the 3’ end can offer efficient protection against snake venom phosphodiesterase I (SVPD) [49]. SVPD is, however, able to completely digest substrates of a scattered arrangement with singular LNAs [50,51]. DNase I is an endonuclease that cleaves single- or double-stranded DNA adjacent to pyrimidines. Remarkably, by using 5’ and 3’ terminal LNA nucleosides together on both strands in a DNA duplex Crinelli *et al.* [52] saw a marked improvement in stability over that observed with the unmodified DNA duplex. Internal modifications did not improve the nuclease resistance further, though. In human serum the increase on DNA oligomer half-life varies with the number of terminal LNA moieties, from 2.5-fold (1 LNA at either end), 11-fold (3 LNA monomers at both ends) to 15-fold (4 LNA monomers at either end) [14]. Further gains can be achieved with mix-mer designs, which have been shown to yield 30-fold improvements in half-life [38]. Together, these reports leave little doubt that modification with LNA offers tremendous advantages regarding stability of therapeutic oligomers.

## 3. Functional Nucleic Acids with LNA

### 3.1. LNA in Aptamers

Aptamers are nucleic acid oligonucleotides, whose sequence allows them to fold into defined tertiary structures that act as ligands and bind their corresponding target molecule with specificity and affinity rivalling that of antibodies. Aptamers have been raised against metal ions, small organic dyes, neurotransmitters, nucleotides, cofactors, amino acids, oligonucleotides, carbohydrates, antibiotics, many proteins, anthrax spores, and live cells. A more comprehensive list is provided elsewhere [53]. The potential uses of aptamers are numerous and include applications in purification, sensors, diagnostics, and therapeutics. Further information can be found in excellent reviews, e.g., [54,55,56] and references highlighted therein. However, to date, only 14 aptamer-based drugs have entered clinical trials [55].

**Figure 2:**
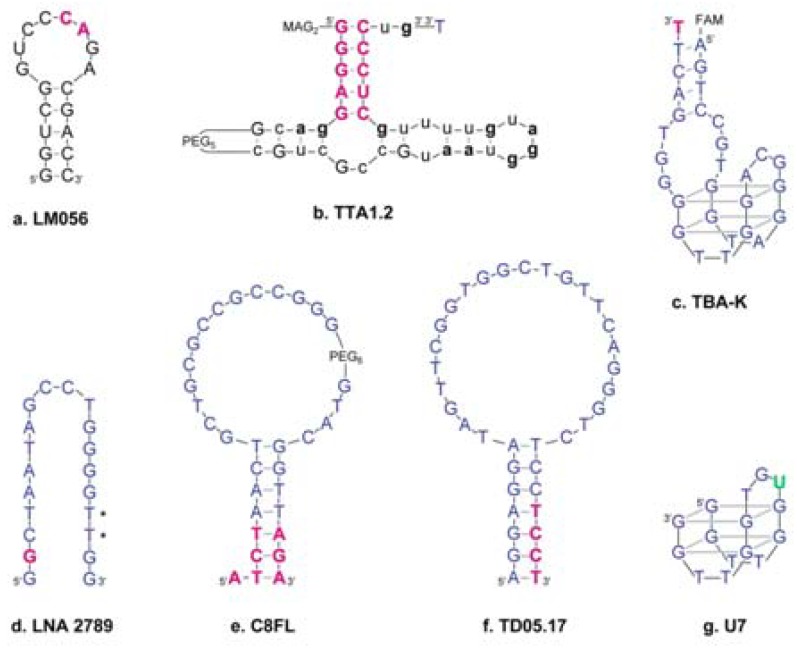
Presumed secondary structures of various modified aptamers. RNA, black; DNA, blue; LNA, purple; UNA, green. (**a**). Aptamer against HIV-1 trans-activation responsive (TAR) element [59]; (**b**). Human Tenascin-C-binding aptamer [61]. Lowercase boldface, 2’-OCH_3_-modification; lowercase non-bold, 2’-F-modification; (**c**). Thrombin-binding aptamer [62], the structure of this aptamer remains elusive. FAM, fluorescein label; (**d**). Avidin-binding aptamer [63]. Asterisks indicate sites that allow for modification with 2’-amino-LNA; (**e**). Aptamer against T-cell leukaemia (CCRF-CEM) cells [64]; (**f**). Aptamer against the mIgM BCR epitope on B-cells [65]; (**g**). Thrombin-binding aptamer modified with UNA [66].

One the first reports of LNA modification to an RNA aptamer was that of the HIV-1 *trans*-activation responsive (TAR) RNA ‘aptamer’ R06 that disrupts the TAT-Tar interaction by forming a ‘kissing loop’ complex with the TAT RNA [57]. As its binding mode consists of base-pairing of six nucleotides in the apical loop to the TAR RNA it can be argued that this is not a true aptamer but a cross-over to the field of antisense oligomers. LNA modifications were placed within these six nucleotides, and only mix-mer designs afforded binding [58]. A complete screening of all possible LNA/2’-O-methyl modifications to the loop later yielded optimized structures with ~20-fold improvements in K_d_ into the sub-nanomolar range (Figure 2a) [59].

The first example of an LNA-modified aptamer not binding through base pairing with the target was with the 2’-F pyrimidine RNA aptamer ‘TTA1’. This 39-mer aptamer forms a three-way junction and binds human glycoprotein Tenascin-C, which resides in the extracellular matrix [60]. LNA moieties were permitted in only one of the aptamer’s supposed three stems, and together with 5’-conjugation to a 2-mercaptoacetylglycyl-glycyl (MAG_2_) chelator for attachment of technetium-99m this lead to increased serum stability and improved tumour uptake and blood retention in tumour-bearing nude mice (Figure 2b) [61].

The first DNA aptamer ever to be identified, the thrombin binding aptamer (TBA) [67], was modified with a single LNA G substitution at the 3’ terminus. This led to decreased thrombin binding [68], and subsequent attempts to introduce LNA to the aptamer’s G-quadruplex structure yielded no improvements over the unmodified aptamer thereby demonstrating how this aptamer depends on an induced fit that is incompatible with LNA [69]. Kasahara *et al.* [62] found that 3’-end capping of a very similar thrombin-binding aptamer [70] with LNA had little effect on binding affinity, though (Figure 2c).

A similar study on an avidin-binding DNA aptamer was recently published by our lab. The previously un-optimized aptamer [71] was truncated from 61 to 21 nucleotides, increasing the K_d_ 10-fold. The aptamer was then screened for various single and dual LNA substitutions, and one substitution restored the K_d_ to that of the original aptamer. 2’-*O*-methyl substitution at this site did not yield the same effect. Further introduction of T-2’-amino-LNA caused only a small increase in K_d_, suggesting that this aptamer was very tolerant towards LNA modifications in general (Figure 2d) [63].

Shangguan *et al.* [64] truncated aptamer ‘sgc8’ against T-cell leukaemia cell line CCRF-CEM while retaining its K_d_. Various LNA substitutions were tested and LNA was accepted in the terminal stem region. This modification, along with replacing a presumed single-stranded region with a polyethylene glycol linker, yielded an aptamer with markedly improved serum stability and a K_d_ ~2-fold higher than that of the original aptamer (Figure 2e).

The most recent example of an LNA-modified aptamer is the modification of DNA aptamer ‘TD05’, which binds a B-cell surface epitope dubbed ‘mIgM BCR’ that is expressed on B-cells and many B-cell lymphomas [72,73]. The aptamer’s stem-loop structure was truncated, causing a near 10-fold increase in target affinity, and linking multiple aptamers together yielded up to 5-fold further improvements. Three arrangements of LNA were evaluated and LNA modification of pyrimidines at the base of the stem gave the best results and offered, as expected, prolonged stability in human serum (Figure 2f) [65].

### 3.2. LNA in Catalytic Nucleic Acids

The term ‘ribozyme’ refers to well-defined RNA structures with the ability to catalyse specific chemical reactions. The first ribozyme to be discovered was a self-splicing RNA [74,75]. Since then, ribozymes have been identified in a number of various biological settings, as well as in *in vitro* experiments [76]. Deoxyribozymes or ‘DNAzymes’ are equivalent catalytic DNA structures obtained by *in vitro* selection, of which the first was an RNA-cleaving Pb^2+^-dependent DNA oligomer [77]. Although the tertiary interactions required for catalysis remain elusive modification with LNA has been investigated.

Catalytic nucleic acids that cleave RNA bind their substrates by Watson-Crick base-pairing and thus seem very well suited to modification with LNA. For instance, the ‘10–23’ DNAzyme [78] was modified to contain two LNA monomers in each of the binding arms, which resulted in much improved cleavage of even a highly structured RNA of 2,904 nucleotides as well as the 17 nucleotide minimal RNA substrate [27,34]. Incorporation of 3–4 LNA moieties into another ‘10-23’ variant yielded similar results against viral RNA and even cleaved otherwise inaccessible targets in highly structured RNA but at the cost of decreased turnover rates [33,36]. A number of studies demonstrating similar modifications to the substrate binding arms have also been published [28.^29^.^30^,^31^,^32^,^34^,^35^,^37^].

**Figure 3:**
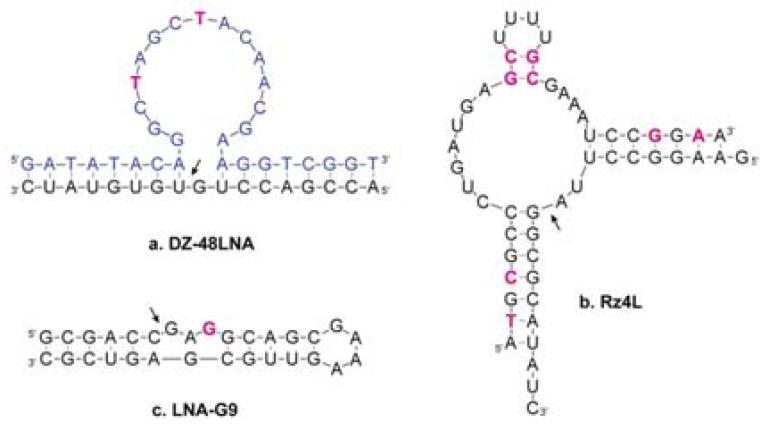
Secondary structures of various modified catalytic nucleic acids. Arrows indicate backbone cleavage sites. RNA, black; DNA, blue; LNA, purple. (**a**). ‘10–23’ DNAzyme, these modifications led to reduced activity [79]; (**b**). Hammerhead ribozyme, the shown modifications led to slightly improved multiple turnover kinetics [81]; (**c**). Leadzyme, this modification increased the activity 20-fold [86].

Beneficial modification of the catalytic cores has proven more difficult. Robaldo *et al.* [79] introduced LNA-T into two positions in the ‘10–23’ core and this drastically reduced the activity of the enzyme (Figure 3a). The purported catalytic stem-loop of the ‘8–17’ DNAzyme [78] has been the target of LNA modification in our lab, but this led to inactivation of the modified DNAzyme [80].

Christiansen *et al.* [81] explored LNA incorporation into the ‘hammerhead’ ribozyme [82] and found that interspersing LNA with DNA in the substrate binding arms increased single turnover rates 20-fold and also improved multiple turnover behaviour. Modifications to ‘stem II’ did not improve the ribozyme, except for one variant that showed a slight improvement in multiple turnover kinetics (Figure 3b). Fedoruk-Wyszomirska *et al.* [83] introduced LNA in one substrate binding arm as well as an auxiliary domain in their hammerhead variant and found that the LNA had no impact on single-turnover rates nor mRNA silencing efficacy in HeLa cells.

Finally, LNA was used to probe the requirements for C3’-endo sugar puckering at three positions in a small, lead-dependent ribozyme [84,85]. This revealed two discrepancies between the inactive ground states recorded by X-ray crystallography and NMR and the catalytically active structure: LNA substitution of a nucleoside believed to be in the C3’-endo pucker caused a 15-fold decrease in self-cleavage activity, whereas LNA substitution at a position believed to exhibit C2’-endo pucker increased the activity 20-fold (Figure 3c) [86].

## 4. Unlocked Nucleic Acids

### 4.1. Properties

Unlocked nucleic acid (UNA) [6,7,87] lacks the C2’-C4’ bond normally found in ribonucleosides (Figure 1), and it is therefore highly flexible. The term ‘unlocked’ hence reflects the structural differences from ‘locked’ nucleic acid [88]. This is also mirrored in the destabilizing effects of UNA-incorporation in duplexes, with up to 12 °C decrease in T_m_ per UNA monomer in RNA:RNA duplexes and 10 °C per UNA monomer in RNA:DNA duplexes [6]. Incubation of a UNA A tri-mer with SVPD or in cell lysate indicates that UNA is also highly resistant to nucleases [89]. UNA monomers have proven very useful in fine-tuning the specificity and potency of siRNA, in particularly in combination with LNA [90,91,92]. It is noteworthy that Bramsen *et al.* [90] found that even highly potent UNA-modified siRNAs did not affect cell viability. Rigorous studies on toxicity have not yet been carried out, however.

### 4.2. UNA in Aptamers

So far, there is only a single published report on UNA-modification to an aptamer: the thrombin-binding DNA aptamer, TBA [67] was systematically substituted with UNA-G or -U monomers. Substitution with UNA-U was tolerated at several positions, and modification to position 7 (Figure 2g) lead to a slightly improved K_d_ (from 103 nM to 78 nM) and a ~3-fold improvement in antithrombin effect, as judged by a fibrin-clot formation assay [66]. The DNA aptamer adopts a G-quadruplex structure, and this places the UNA-U monomer in the central loop. However, structural data on the precise conformation and orientation of the protein-bound aptamer remain ambiguous [93,94,95] and preclude any precise rationalizations on the effects conferred by the UNA on this particular aptamer.

## 5. *De Novo* Selection of Modified Nucleic Acid Structures

Synthetic functional nucleic acids are identified through an iterative technique known as *in vitro* selection [96,97,98,99]. The classic approach involves enriching a sequence pool for members with the desired activity by repeated incubation, amplification, and pool re-generation. Finally, the pool is sequenced. By using a pool of oligomers containing modified nucleosides it is possible to realize functional nucleic acid structures that natively contain these modifications. The use of such nucleosides in classical *in vitro* selection requires that they are tolerated by the enzymes used for amplification and regeneration of the pool [100]. To date, LNA and UNA nucleotides have only been used post-selection. This is probably due to the lack of the appropriate methods for maintaining LNA or UNA-modified pools over successive selection rounds. Phosphoramidite derivatives of all standard nucleobases have been introduced [6,7,101,102,103], thus making the initial synthesis of such libraries a relatively straightforward process; the lower coupling efficiencies of LNA and UNA should be taken into account, however, and may disfavour synthesis of sequences containing consecutive modifications. Reading of LNA-modified templates, as well as incorporation of triphosphates LNA TTP, LNA ATP, LNA GTP, and LNA 5-methyl-CTP, has been demonstrated with Taq [104], Vent(exo-) [104], Phusion High-Fidelity [104,105,106], 9° N_m_ [107,108], and KOD [104,109,110] DNA polymerases, as well as T7 RNA polymerase [107]. The most promising results published so far have been the successful PCR amplification of a 50-nucleotide template while incorporating LNA A at 9 sites [109] and 21 successive incorporations of all LNA nucleosides [110].

Applying LNA to the classical *in vitro* selection setup with many rounds of selection and regeneration remains non-trivial (our unpublished results), and other methods might therefore be considered for selection of LNA-containing structures. Selection by capillary electrophoresis (CE) or ‘non-equilibrium capillary electrophoresis of equilibrium mixtures’ (NECEEM) separates non-binding and binding members in solution and can yield aptamers in as few as 1-4 rounds [111,112,113,114,115,116,117] and may even alleviate the need for pool amplification [112,118]. As only a few rounds of selection are required with CE, the pools used rarely converge [113,114,115,116]. This is seen as an advantage though, as it indicates that larger sequence spaces are explored with this technique.

In another method the library is synthesized directly onto small beads by a split-and-mix technique so each bead becomes coated in a unique sequence. The library is then screened for beads that retain the (labelled) target, and this approach has been used for finding phosphorothioate [119] or iso-guanine-modified aptamers [67]. The technique is, however, limited by the very small size of the libraries, typically containing only a few thousand unique members. 

The enzymatic steps required for pool re-generation and sequencing can be avoided altogether by employing array-based selection. Microarrays have been used for assessing various mutations in a known aptamer [120], and this method also seems well suited for screening for activity of functional nucleic acids modified post-selection. More interestingly, the technique dubbed ‘closed loop aptameric directed evolution’ (CLADE) [121] that uses array-based libraries in combination with in silico evolution has been used to generate aptamers from starting pools of 5,500 [121], 46,000 [122], or 150,000 [123] unique members. The initial array seeks to cover a large sequence space, and experimental binding data are then combined to mutate selected members for the next generation array. This approach has yielded aptamers in 4–9 selection rounds and seems suitable for even highly modified libraries. It therefore seems likely that the future application of LNA and UNA in functional acids may be based on either of these inventive approaches.

## 6. Conclusions

In conclusion, the application of LNA and UNA in functional nucleic acids is still very promising and definitely worth pursuing. The same goes for other LNA derivatives such as amino-LNA [124], which has not been covered by this review. The development of efficient protocols for enzymatic incorporation of modified nucleotides and reading/copying of modified oligonucleotides is reckoned important, specifically for selection of aptamers spiked with LNA, which are predicted to play an important role in diagnostics and biotechnology in the future. As summarized in this review, there are now numerous studies supporting the usefulness of the specific features of LNA and UNA in the field of RNA therapeutics, including aptamers, siRNAs, microRNAs and antagomirs, most of them working by hybridization as a sort of antisense oligonucleotides. *In vitro* selection of function nucleic acids with nucleoside modifications has been very successful with 2’-F pyrimidines and subsequent substitution with 2’-O-methylated purines. Nonetheless, LNA remains a solid candidate for post-selection modification of stem regions and termini, where it offers nuclease resistance and can improve conformational stability. The presence of even a few modifications can also have a large impact on specificity, off-target effects, structure, stability and clearance. Even though the optimal employment of the novel UNA still remains to be seen, it is now largely up to companies to take advantage of both of these nucleoside analogues and develop them for commercial usage.
